# Photodynamic Inactivation of Bovine Coronavirus with the Photosensitizer Toluidine Blue O

**DOI:** 10.3390/v16010048

**Published:** 2023-12-27

**Authors:** Maya Margaritova Zaharieva, Pelagia Foka, Eirini Karamichali, Alexander Dimitrov Kroumov, Stanislav Philipov, Yana Ilieva, Tanya Chan Kim, Petar Podlesniy, Yordan Manasiev, Vesselin Kussovski, Urania Georgopoulou, Hristo Miladinov Najdenski

**Affiliations:** 1 Department of Infectious Microbiology, The Stephan Angeloff Institute of Microbiology, Bulgarian Academy of Sciences, 26 Acad. G. Bonchev Str., 1000 Sofia, Bulgaria; zaharieva26@yahoo.com (M.M.Z.); adkrumov@gmail.com (A.D.K.); illievayana@gmail.com (Y.I.); tanya_85@abv.bg (T.C.K.); vkussovski@gmail.com (V.K.); 2 Department of Microbiology, Laboratory of Molecular Virology, Hellenic Institute Pasteur, Vasilissis Sofias 127, 11521 Athens, Greece; pfoka@pasteur.gr (P.F.); eirinik@pasteur.gr (E.K.); 3 Chair Human Anatomy, Histology, General and Clinical Pathology and Forensic Medicine, Faculty of Medicine, Hospital Lozenetz, Sofia University “St. Kliment Ohridski”, 2 Kozyak Str., 1407 Sofia, Bulgaria; stanislav_philipov@abv.bg; 4 Institute of Biomedical Research of Barcelona, CSIC, Rosselló, 161, 7ª Planta, 08036 Barcelona, Spain; ppodlesniy@gmail.com; 5 Evgeni Budevski Institute of Electrochemistry and Energy Systems, Bulgarian Academy of Sciences, 1113 Sofia, Bulgaria; manasiev_jo@abv.bg

**Keywords:** photosensitizers, toluidine blue O, light irradiation, bovine coronavirus, antiviral activity

## Abstract

Coronaviruses (CoVs) belong to the group of enveloped positive-sense single-strand RNA viruses and are causative agents of respiratory, gastro-intestinal, and central nervous systems diseases in many host species, i.e., birds, mammals, and humans. Beta-CoVs revealed a great potential to cross the barrier between species by causing three epidemics/pandemics among humans in the 21st century. Considering the urgent need for powerful antiviral agents for decontamination, prevention, and treatment of BCoV infections, we turned our attention to the possibility of photodynamic inactivation with photosensitizers in combination with light irradiation. In the present study, we evaluated, for the first time, the antiviral activity of toluidine blue O (TBO) against Beta-coronavirus 1 (BCoV) in comparison to methylene blue (MB). First, we determined the in vitro cytotoxicity of MB and TBO on the Madin–Darby bovine kidney (MDBK) cell line with ISO10993-5/Annex C. Thereafter, BCoV was propagated in MDBK cells, and the virus titer was measured with digital droplet PCR, TCID_50_ assay and plaque assay. The antiviral activity of non-toxic concentrations of TBO was estimated using the direct inactivation approach. All effects were calculated in MAPLE 15^®^ mathematical software by developing programs for non-linear modeling and response surface analysis. The median inhibitory concentration (*IC*_50_) of TBO after 72 h of incubation in MDBK cells was 0.85 µM. The antiviral activity of TBO after the direct inactivation of BCoV (*MOI* = 1) was significantly stronger than that of MB. The median effective concentration (EC_50_) of TBO was 0.005 µM. The cytopathic effect decreased in a concentration-dependent manner, from 0.0025 to 0.01 µM, and disappeared fully at concentrations between 0.02 and 0.3 µM of TBO. The number of virus particles also decreased, depending on the concentration applied, as proven by ddPCR analysis. In conclusion, TBO exhibits significant potential for direct inactivation of BCoV in vitro, with a very high selectivity index, and should be subjected to further investigation, aiming at its application in veterinary and/or human medical practice.

## 1. Introduction

Coronaviruses (CoVs) are well-known to the scientific community, particularly among veterinarians, as they can cause a wide range of diseases, mainly affecting the respiratory, gastro-intestinal, and central nervous systems in a large number of host species, from birds to mammals, including humans. CoVs have become the major pathogens of emerging respiratory disease outbreaks in the 21st century. In the last twenty years, three viral epidemics/pandemics among humans caused by CoVs have been recorded: (1) the severe acute respiratory syndrome coronavirus (SARS-CoV, from 2002 to 2003), which caused a large-scale epidemic beginning in China and spread to twenty-six countries, resulting in approximately 8000 cases and 800 deaths; (2) the Middle East respiratory syndrome coronavirus (MERS-CoV), first identified in Saudi Arabia in 2012, characterized by approximately 2500 cases and still causing disease sporadically, and (3) a novel very contagious Beta-coronavirus (bCoV), named the severe acute respiratory syndrome coronavirus 2 (SARS-CoV-2), which emerged first in Wuhan (China) and spread quickly all over the world. So far, the pandemic has affected all areas of human life, especially healthcare, with over 500 million confirmed infections and nearly 6.3 million infection-related deaths reported globally to the World Health Organization (WHO) [1]. The 45 identified CoVs species belong to the group of the enveloped single-strand positive-sense RNA viruses classified into four genera—Alpha, Beta, Gamma, and Delta—within the *Coronaviridae* family, order *Nidovirales* [2]. The Alpha- and Beta-genera originate from mammals, in particular bats, whereas the reservoirs of the Gamma- and Delta-genera are pigs and birds [3].

While the above mentioned subgenera of the Beta-coronaviruses cause diseases in humans, with varying degrees of infectious potential, such as lower respiratory tract infections (HCoV-NL63 and HCoV-HKU1) or severe pneumonia (SARS-CoV, Middle East respiratory syndrome coronavirus MERS-CoV, and SARS-CoV-2) [4], other species are fatal for farm animals, leading to serious economic losses [5]. The bovine coronavirus (BCoV) is not only a causative agent of gastrointestinal and respiratory diseases with often lethal outcome in cattle, but it is also characterized by close genetic features with the human coronavirus type OC43. The importance of the BCoV for public health is supported by the fact that a coronavirus related to BCoV has been isolated from human feces of patients with diarrhea [6].

Despite the emergence of effective vaccines, the development of broad-spectrum antiviral treatments remains a significant challenge, in which antimicrobial photodynamic therapy (PDT) may play a role, especially at early stages of infection or in prevention and disinfection. In the light of spreading antibiotic resistance and the rise of new infections, the photodynamic inactivation (PDI) of microbes is gaining considerable attention as a promising technique for inactivating bacteria, viruses, fungi, and protozoa, as well as for the local treatment of infections [7,8,9,10]. The PDI of viruses shares the general action mechanism of photodynamic applications: the irradiation of a dye with light and the subsequent generation of reactive oxygen species (ROS), which are the effective phototoxic agents, damaging virus targets by reacting with viral nucleic acids, lipids, and proteins. Interestingly, a light-independent antiviral activity has also been found for some of these dyes. In the medical area, currently two fields stand out in which the PDI of viruses has found broader application: the purification of blood products and the treatment of human papilloma virus manifestations. For inactivation of biological targets, PDI needs three conditions: (1) the so-called light-sensitive compound (photosensitizer—PS); (2) a light source for activation of the PS; and (3) molecular oxygen. After the appropriate irradiation of the photosensitizer directed at the pathogen, the generation of singlet oxygen and other reactive oxygen species occurs, damaging the target. The photodynamic mode of action is based on two photochemical reactions—type I and type II. The type I mechanism functions via electron/proton relocation from an excited state of a photosensitizer [11]. The type II mechanism of energy involves transfer from the excited triplet state of a photosensitizer to triplet oxygen (^3^O_2_), resulting in the formation of singlet oxygen (^1^O_2_). The formed reactive species oxidize and damage subcellular structures and macromolecules, leading to cell death.

The group of pharmacologically active PS includes hematoporphyrin derivatives, phenothiazines, cyanines, phthalocyanines, and chlorines [12]. The enveloped viruses, such as CoVs, represent a convenient target for the PS mode of action, mainly because the virus envelope proteins and unsaturated lipids can be easily oxidized with reactive oxygen species and free radicals. It is well known that lipid peroxidation affects not only the functioning of the membrane, but also the activity of the surrounding proteins [13]. The PDI of viruses has found interest in such diverse areas as water and surface decontamination and biosafety. The review of Wiehe et al., 2019 [14] gives comprehensive information on the mechanisms and targets for viral PDI and the structure and activity of a large groups of PS for viral PDI, such as curcumins, perylenequinones (hypericin, hypocrellins, and related compounds), metal oxides and other inorganic materials, fullerenes and carbon materials, porphyrins and porphyrinoids (porphyrins, chlorins, phthalocyanines), riboflavin, psoralens, phenothiazines, and methylene blue, rose bengal, cyanine dyes, rhodamine B, and derivatives such as octadecyl rhodamine B (‘R18), as well as multicomponent plant extracts.

Various metal phthalocyanines have been studied for their capacity for photodynamic inhibitory effects on different viruses. Two newly synthesized water-soluble phthalocyanine Zn(II) complexes with different charges—cationic methylpyridyloxy-substituted Zn(II)-phthalocyanine (ZnPcMe) and anionic sulfophenoxy-substituted Zn(II)-phthalocyanine (ZnPcS)—were used for PDI of DNA-enveloped viruses such as herpes simplex virus type 1 (HSV-1) and vaccinia virus (VV), RNA-enveloped viruses such as bovine viral diarrhea virus (BVDV) and Newcastle disease virus (NDV), and two naked viruses (the enterovirus Coxsackie B1, a RNA-containing virus, and human adenovirus 5, a DNA virus). Both phthalocyanine complexes showed an identical marked virucidal effect against herpes simplex virus type 1 at an irradiation lasting 5 or 20 min (Δlog = 3.0 and 4.0, respectively). This effect was weaker towards vaccinia virus, wherein Δlog = 1.8 for ZnPcMe and 2.0 for ZnPcS. Bovine viral diarrhea virus manifested a pronounced sensitivity to ZnPcS at 5 and 20 min irradiation (Δlog = 5.8 and 5.3, respectively) and only a moderate one to ZnPcMe (Δlog = 1.8). The complexes did not inactivate Newcastle disease virus, Coxsackievirus B1, and human adenovirus type 5 [15]. Three other photosensitizing phthalocyanine derivatives were tested for antiviral activity with PDI towards the following coated and non-enveloped viruses: bovine viral diarrhea virus (BVDV), influenza virus A (H3N2), poliovirus type 1 (PV-1), and human adenovirus type 5 (HAdV5). Virucidal and irradiation effects were registered toward BVDV by octa-methylpyridyloxy-substituted Ga phthalocyanine (Ga8), whereas only tetra-methylpyridyloxy-substituted Ga phthalocyanine (Ga4) exhibited a remarkable PDI. No effect was determined towards influenza A virus. In contrast, the Ga4 and Ga8 exhibited remarkable PDI potential on naked viruses, especially on HAdV5, revealing combined virucidal and irradiation effects [16].

Previous investigations on porphyrinoids have highlighted their effective inactivation of enveloped viruses, such as HIV [17] and influenza viruses [18]. One of these water-soluble cationic PS, octakis (cholinyl) zinc phthalocyanine (Zn-PcChol8+), completely destroyed the infectivity of SARS-CoV-2 in combination with far red light irradiation [19]. The enveloped avian H5N8 influenza virus was also sensitive to PDI with Zn-PcChol8+. The mode of action of this PS was revealed by transmission electron microscopy (TEM). The pictures showed a loss of the H5N8 virus membrane surface glycoproteins, which caused complete disintegration of the virus envelope [18].

The phenothiazines methylene blue (MB) and toluidine blue O (TBO) are among the most studied PS for bacterial biofilm inactivation [20]. Both are amphiphilic and can be used against Gram-positive and Gram-negative bacteria present in endodontic infections. Furthermore, MB has been shown to possess antiviral activity, as it fully protected Vero E6 cells from infection with SARS-CoV-2 after irradiation with a wave length of λ = 662 nm [21].

The other phenothiazine, TBO, is an amphiphilic, positively charged compound with low molecular weight and peak absorption at 635 nm, which exhibits a great potential for photooxidation. TBO generates high quantum yields of singlet oxygen leading to oxidative damage of the cytoplasmic membrane, different membrane proteins, and bacterial enzymes during the photochemical process [22]. It has also been reported that the PDI active concentration range of TBO is non-toxic for human cells [23], which makes TBO a promising antibacterial agent against *Staphylococcus aureus*, as long as bacterial photoinactivation requires a lower concentration of PS and short exposure to light [24]. TBO and MB have also proven their bactericidal effects against root canal infections caused by a mixture of Gram-positive and Gram-negative bacteria by eradicating successfully *Enterococcus faecalis* and other bacteria from root canals [25]. Kömerik et al. [26] showed that TBO-mediated bactericidal photosensitization of *Porphyromonas gingivalis* is successful in vivo and this results in decreased bone loss. This finding suggesting the PDT may be a useful alternative approach for the treatment of periodontitis. Currently available to dentists is the commercial product FotoSan 630 (CMS Dental, Copenhagen, Denmark) with an LED lamp emitting light in the red spectrum, with a power peak at 630 nm and an output intensity of 2000–4000 mw/cm^2^. Further, Najm et al. [27] estimated the effectiveness of PDT with TBO in a combination with light-emitting diode (LED) for treatment of cutaneous leishmaniasis. The study resulted in significant decreases of the *Leishmania major* promastigotes and intra-cellular amastigotes viability.

Antiviral PDI has not yet been accepted as an established method in the clinical setting and, to date, it is mostly limited to topical applications, such as oral decontamination [28] and treatment of orofacial manifestations [29] in patients suffering from COVID-19. In addition, PSs have a good potential to be applied for the disinfection of surfaces in medical premises and buildings. The data in the scientific literature on the antiviral activity of certain cationic PSs such as TBO are still scanty. In this regard, the present study aims to evaluate, for the first time, the antiviral activity of TBO on BCoV, in comparison to the well-studied PS MB, using the direct inactivation approach.

## 2. Materials and Methods

### 2.1. Chemicals and Reagents

The following media, enzymes, and sera used for cell culturing were purchased from Capricorn Scientific, Ebsdorfergrund, Germany: Minimal Essential Medium (MEM) with Earle’s salts (#MEM-A), fetal bovine serum (#FBS-HI-12A), pen/strep 100× (#PS-B), stable L-glutamine, non-essential amino acids (NEAA), and Accutase^®^ (#ACC-1B). The chemicals 3-(4,5-dimethylthiazolyl-2)-2,5-diphenyltetrazolium bromide (MTT, #M2128-1G), Sodium pyruvate (#S8636), and Dulbecco’s phosphate-buffered saline (PBS, #D8537) were products of Merck (Sigma-Aldrich, Steinheim, Germany). Methylene blue (MB, #PHR3838) and toluidine blue O (TBO, #198161) were purchased from Merck (Darmstadt, Germany).

### 2.2. Cultivation of MDBK Cells

The Madin–Darby bovine kidney cell line MDBK (NBL-1, #600396) was purchased from CLS Cell Lines Service (GmbH, Eppelheim, Germany). Cells were maintained in sterile culture flasks in MEM, supplemented with 2 mM GlutaMAX™, 0.1 mM non-essential amino acids, 1 mM sodium pyruvate, and 10% (*v*/*v*) FBS. The culture conditions were controlled in a CO_2_ incubator (Panasonic MCO-18AC, Panasonic Healthcare co., Ltd., Oizumi-Machi, Japan) as follows: 37 °C, 5% (*v*/*v*) CO_2_ and humidified atmosphere. Cells were split every 3 days at a ratio of 1:4 using PBS and Accutase^®^ according to the protocol of the biobank.

### 2.3. Evaluation of In Vitro Cytotoxicity with the MTT-Dye Assay

The cytotoxicity of MB and TBO was evaluated according to Annex C, ISO 10993-5 [30,31]. The results were used to calculate the median inhibitory (*IC*_50_) and the maximum non-toxic concentrations (MNCs) of both compounds on MDBK cells in the absence of BCoV. *IC*_50_ was the concentration required to reduce the dehydrogenase activity or to induce visible morphological changes in 50% of cells. The MNC was defined as the minimum dilution of fraction that did not cause toxic effects or death of the treated cells. Briefly, the cells were plated in 96-well plates at a density of 0.135 × 10^6^ cells/mL, with 100 µL/well, and cultured for 24 h to enter the log phase of their growth. Thereafter, cells were treated with 10 concentrations of MB or TB in twofold serial dilutions from 40 µM to 0.078 µM. The treated cells were incubated for 24, 48, and 72 h under controlled conditions in CO_2_ incubator (see description above). The cell viability was measured at the end of each incubation period, whereby 10 µL of MTT solution (5 mg/mL) was added to each well and the plates were kept at 37 °C for 2 h. The formazan crystals formed in the surviving cells were dissolved with 100 μL/well of 2-propanol after the aspiration of the supernatant. The absorbance was measured at λ = 540 nm (reference filter at 690 nm) on a microplate reader ELx800 (BioTek Instruments, Inc., Winooski, VT, USA).

### 2.4. Determination of MDBK Specific Growth Rate

Briefly, cells were plated at 6 different concentrations—0.04, 0.06, 0.08, 0.1, 0.125, and 0.15 × 10^6^/mL—in 96-well sterile plates, in a volume of 100 µL/well and 16 wells/concentration. The cell absorbance was measured every 24 h up to the 96th hour from cell plating. The MTT-dye reduction assay (ISO 10993-5/2006, Annex C [30,31]) was applied for measurement of the cell viability, as described above. The specific growth rate (SGR) of the cells was calculated in MAPLE 15^®^ mathematical software (Maplesoft, a division of Waterloo MAPLE Inc., Ontario, CA, USA) according to the well-known mass balance equation of the batch process.

1. Biomass balance:

(1) $$\frac{dX}{dt}=\mu \times X$$

(2) $$\int_{X1}^{X2}\frac{dX}{X}=\int_{t1}^{t2}\mu dt$$

2. Solution to the differential equation:

(3) $$\ln\left(X_{2}\right)-\ln\left(X_{1}\right)=\mu \times \left(t_{2}-t_{1}\right)$$

3. After rearrangement of the form of the Equation (3), SGR is obtained as follows:

(4) $$\mu =\frac{\ln\left(X_{2}\right)-\ln\left(X_{1}\right)}{\left(t_{2}-t_{1}\right)}$$

where *t*_1_ and *t*_2_ are the time points of cultivation in [h]; *X*_1_ and *X*_2_ are the biomass concentrations at the time points *t*_1_ and *t*_2_ in [g/L]; and *μ* is the specific growth rate (SGR) in [h^−1^]. In order to compute the maximum SGR for the given cultivation conditions, time can be chosen from zero up to the stationary phase of cell growth or divided into any smaller intervals in the logarithmic phase of the growth curve. The doubling time (*t_d_*) was calculated from Equation (4) as follows:

(5) $$t_{d}=\frac{\ln(\frac{2\cdot X_{1}}{X_{1}})}{\mu }=\frac{\ln(2)}{\mu }=\frac{0.69}{\mu }$$

where 2·*X*_1_ is equal to *X*_2_ and *µ* is the specific growth rate. The doubling time was calculated for each of the 6 different initial cell concentrations.

### 2.5. Mathematical Model for Calculation of Median Inhibitory and Maximal Non-Toxic Concentration

All effects were calculated using MAPLE 15^®^ mathematical software by developing programs for non-linear modeling and response surface analysis (RSA) of the experimental data points. The median inhibitory concentration (*IC*_50_) was calculated with MAPLE 15^®^ mathematical software using a non-linear mathematical model based on Chou and Talaly [32,33]. For this, a non-linear regression procedure was coded in MAPLE 15^®^ software of symbolic mathematics based on the weighted least squares statistical criterion as an objective function of the search. In order to minimize the sum of weighted squares and find the estimates of best-fitting parameter values, we used a numerical optimization algorithm. The median-dose model applied for calculation of “*IC*_50_” and “*m*” is as follows:

(6) FaFu=DoseDmm,

where *F_a_* is the affected fraction; *F_u_*—the unaffected fraction (1 *− F_a_*) = *F_u_*; *Dose*—applied compound concentration; *D_m_*—median-effect concentration (in our study *D_m_* = *IC*_50_); and *m*—a *hillslope* of the median-effect plot (for *m* = 1 the curve is hyperbolic; for *m* > 1, sigmoidal; for *m* < 1, negative (flat) sigmoidal). A response surface analysis (RSA) methodology was used to reveal the predictive power of the model as a function of the parameters “*IC*_50_” and “*m*”. The range of the parameters’ value changes was determined in RSA 3D plots based on the standard deviation of the “*IC*_50_” and “*m*” values obtained during the statistical evaluation of the experimental data with GraphPad Prism software. The latter software was used to calculate the maximal non-toxic concentrations from a non-linear curve based on a [log(inhibitor) vs. normalized response—variable slope] model:

(7) $$Y=\frac{100}{1+10^{(\log{IC}_{50}-X)\times hillslope}}.$$

### 2.6. Propagation of Bovine Coronavirus in MDBK Cells

MDBK cells were infected with the bovine coronavirus strain “S379 Riems” (022V-04370 Beta-coronavirus 1, FLI, WOAH Collaborating Centre for Zoonoses in Europe, Greifswald—Insel Riems, Germany). The viral titer of the stock was 1.47 × 10^7^ (TCID_50_/mL) according to the data sheet of the delivery organization. MDBK cells were plated in a 25 cm^2^ sterile cell culture flask (Corning, Glendale, Arizona, USA) at a concentration of 0.45 × 10^6^/mL in 5 mL of complete culture medium and incubated for 24 h until reaching approximately 90% confluency. Thereafter, the medium was changed to 2 mL of MEM supplemented with 2% (*v*/*v*) FBS, and the cell monolayer was infected with BCoV at a multiplicity of infection (*MOI*) 1 (see Equation (1)). The cells were incubated for 3 h and the medium was replaced with 4 mL of the complete culture medium containing 10% (*v*/*v*) FBS. The cytopathic effect (CPE) was observed 48 h after the start of the infection under an inverted microscope. The supernatant was collected and used for a second-round infection of MDBK cells plated in a 75 cm^2^ sterile culture flask in the corresponding cell density. After 3 h of incubation, the medium was discarded, and 7 mL complete culture medium was added to the flask. Cells were cultured for 48 h until the appearance of the CPE, and the medium with the cells was collected and centrifuged for 10 min at 500× *g*. The supernatant was aliquoted in cryovials (1 mL/tube) and stored at −80 °C. The aliquots were used for the subsequent experiments after determination of the *viral titer* with ddPCR, TCID_50_, and plaque assays. The amount of virus stock needed for the first-round of infection of MDBK cells was calculated according to the following equation:

(8) $$V\;\left[mL\right]=\frac{number\;of\;cells\times number\;of\;culture\;flasks\times MOI}{viral\;titer}$$

where *V* is the volume of virus stock solution needed for the infection of a certain *number of cells* in a certain *number of culture flasks* with the chosen multiplicity of infection.

### 2.7. Determination of the Bovine Coronavirus Titer with Droplet Digital (ddPCR)

For the isolation of the bovine coronavirus RNA, we used the NucleoSpin RNA virus kit (Marcherey-Nagle GmbH & Co. KG, Deuren, Germany) according to the instructions of the producer. The concentration of the obtained RNA was quantified using the NanoDrop™ Lite spectrophotometer (ThermoFisher Scientific, Waltham, MA, USA). One ng of viral RNA was reverse transcribed with the PrimeScriptTM Reverse Transcriptase (TaKaRa Bio Inc., Shiga, Japan) and the produced cDNA was used for the determination of the number of virus particles in the stock with the ddPCR. Briefly, 7 tenfold serial dilutions (from 10^−1^ to 10^−7^) were prepared from the cDNA. Thereafter, 9 µL of each dilution were used for the ddPCR. The latter was prepared using the ddPCR^TM^ Supermix for Probes (Bio-Rad Laboratories Inc., Hercules, California, USA), as suggested by the manufacturer. The primer/probe set targeted the nucleocapsid gene of the BCoV and consisted of the following sequences: forward primer 5′-GGACCCAAGTAGCGATGAG-3′; reverse primer 5′-GACCTTCCTGAGCCTTCAATA-3′ and probe 6-FAM-5′-ATTCCGACTAGGTTTCCGCCTGG-3′-BHQ1 [34]. The primers and probe were added to the master mix in final concentrations of 900 nM and 200 nM, respectively, to a final volume of 20 µL. Two replicates of each dilution and a negative control (PCR water) were prepared. The temperature protocol was as follows: initial denaturation at 95 °C (10 min), 40 cycles of denaturation at 94 °C (30 s), annealing and extension at 57 °C (1 min), enzyme deactivation at 98 °C (10 min) and reaction end at 4 °C (∞). The ramp rate between temperature changes was 2 °C/s. The concentration of the viral particles in the stock from which RNA was isolated was calculated having in mind all subsequent dilutions during isolation, reverse transcription and ddPCR reaction, which were as follows: 150 µL supernatant containing virus particles ➝ 50 µL RNA yields ➝ 1 ng RNA for 20 µL master mix for reverse transcription ➝ 9 µL cDNA or 20 µL ddPCR. This method was also used for enumeration of the virus particles in samples exposed to TBO, whereby 200 µL of the supernatant was used for RNA isolation.

### 2.8. Determination of the Bovine Coronavirus Titer by TCID_50_ Assay

For determination of the virus titer with the TCID_50_ assay [35,36], the day prior to infection, 0.15 × 10^6^/mL MDBK cells were seeded in a 96-well plate in a 100 µL/well. On the day of infection, 8 serial 10-fold dilutions from 10^−1^ to 10^−8^ of the viral stock (propagated as described above) were prepared in MEM, complemented with 2% (*v*/*v*) FBS, and used to infect the monolayer of MDBK cells. Ten replicates were prepared from each dilution. Twelve wells were used as no-virus control. Following a 3 h incubation, the medium was replaced with regular 10% (*v*/*v*) FBS MEM. The plate was incubated further for 96 h and observed daily to monitor the development of CPE under an inverted optical microscope. On day four, the number of wells with CPE were evaluated and recorded. The MTT-dye assay was applied to determine cell viability, as described in ISO 10993-5/2006 [30,31]. Viral titers, expressed as TCID_50_/mL, were calculated according to both Reed-Muench [36,37,38] and Spearman-Kärber [39,40], methods.

### 2.9. Determination of the Bovine Coronavirus Titer and Antiviral Activity of Photosensitizers by Plaque Assay

The viral titer was also determined via the plaque assay. Briefly, the day before the infection, 0.3 × 10^6^ cells/well were seeded in a 12-well plate and incubated overnight (37 °C, maximal humidity, 5% CO_2_) to allow the monolayer to reach approximately 90% of cell density. On the day of infection, 10 serial 10-fold dilutions from 10^−1^ to 10^−10^ of the viral stock (propagated as described above) were prepared in MEM complemented with 2% (*v*/*v*) FBS. The medium was removed from the plate and replaced by 1 mL of each dilution. Following 3 h of incubation, the viral inoculum was aspirated from the wells and replaced with 1 mL regular 10% (*v*/*v*) FBS MEM culture medium. The plate was further incubated for 48 h at 37 °C. After incubation, the cells were washed with PBS and fixed with methanol (10 min at RT). Upon methanol removal, the monolayer was stained with 1% (*w*/*v*) crystal violet (20 min at RT), washed twice with distilled water, and dried. The plaques were counted under an inverted biological microscope Boeco BIB-100 (Boeckel GmbH + Co, Hamburg, Germany) with 200× magnification.

For evaluation of the antiviral activity of the photosensitizers investigated in this study, the cells treated with viral inoculum, as well as the negative control and samples treated with MB and TBO were observed daily for CPE. The morphological changes were documented in live culture under an inverted biological microscope (described above) with 100× magnification. The cells were examined by an expert pathologist for specific morphological changes, including external cell morphology, cell detachment, presence of permissive cells, and plaque-forming units.

### 2.10. Determination of the Antiviral Activity of the Photosensitizers

The potential of MB and TBO for direct inactivation of the bovine coronavirus was determined after light irradiation with a LED (light-emitting diode) device (produced by ELO Ltd., Sofia, Bulgaria). The device contains 25 super bright diodes at 635 nm with spectral half width of about 20 nm. The effect of the compounds was evaluated microscopically regarding the CPE and confirmed biochemically with the MTT-dye reduction assay according to ISO 10993-5/2006, Annex C, with some modifications [30,31]. Briefly, 0.135 × 10^6^ cells/mL cells were plated in 96-well plates in a volume of 100 µL/well. After 24 h, the medium was aspirated and replaced with 100 µL/well viral inoculum containing different concentrations of MB or TBO. The viral inoculum was prepared as follows: eight different concentrations of each compound ranging from 0.0025 µM up to 0.3 µM in 2% (*v*/*v*) FBS medium MEM with bovine coronavirus at *MOI* = 1 were aliquoted in a 96-well plate in eight replicates (100 µL/well each). Half of the samples of each concentration, including both controls, were irradiated with a light dose of 54 J/cm^2^ for MB (680 nm) and 99 J/cm^2^ for TBO (635 nm). Viral inoculum without compounds and viral inoculum without culture medium served as positive and negative controls, respectively. The plates were incubated for 3 h at 37 °C in a 5% CO_2_ incubator with humidified atmosphere. Thereafter, the supernatant in all wells was aspirated, and 100 µL/well regular 10% (*v*/*v*) FBS medium MEM was added. The plates were further incubated for 4 days, and the cells were evaluated daily microscopically for morphological changes and signs of CPE. At the end of the incubation period, microscopic pictures of treated and untreated cells were taken. Cell viability was measured with the MTT-dye reduction assay, as described above. The maximal effective concentration 50% (EC_50_) was expressed as the concentration that achieved 50% protection of cells from the virus-induced death. The EC_50_ was calculated with MAPLE 15^®^ software using a non-linear mathematical model. The selectivity index (SI) was determined as the ratio between *IC*_50_ in MDBK cells and EC_50_ necessary for 50% direct BCoV inactivation [41].

### 2.11. Statistical Evaluation

The statistical evaluation of the experimental data was performed with GraphPad Prism software (Version 6.00, for Windows, GraphPad Software, La Jolla, CA, USA). Each experiment was performed in triplicate. Minimum three samples for each concentration, the positive, negative, and untreated controls were prepared. Data are presented as mean ± SD. One-way ANOVA analysis of variance was applied to compare two groups of samples. A value of *p* < 0.05 was considered statistically significant.

## 3. Results

### 3.1. Specific Growth Rate of MDBK Cells

The SGR of MDBK cells was evaluated after measuring the absorbance of the cells with the MTT dye-assay every 24 h, up to the 96th hour of the culture. The duration of the culture was chosen based on the experiments planed for evaluation of the antiviral activity. The result obtained from this experiment was used for determination of the doubling time of the cells. This information is needed for estimation of the initial cell density so that the desired cell confluence is reached at the time of viral inoculation. The average absorbance values for each cell concentration and incubation period, as well as the corresponding doubling times, are given in Table 1. As shown, an increase in cell density was observed for each set of data. The absorbance increase rate depended on the initial cell number/mL.

**Figure 1:**
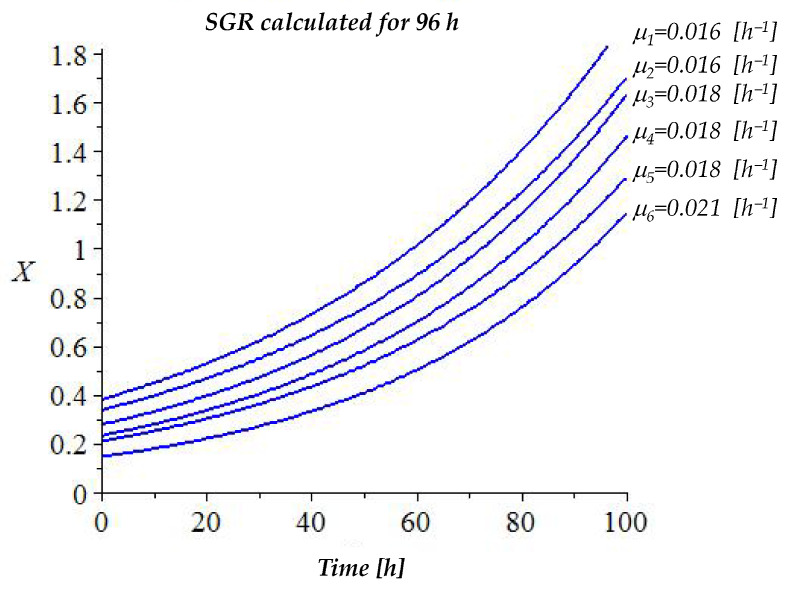
Specific growth rate of MDBK cells at different initial seeding concentrations. Legend: *X* = absorbance of the cells in culture.

Based on the data from Table 1, curves were plotted for each initial cell concentration. The graph is presented in Figure 1. Based on equations 1÷4, the SGR constant μ was calculated in MAPLE 15^®^ software for each initial cell concentration and the adjacent data set—the values are given in Figure 1 beside the curves. The SGR constants were used for calculation of the doubling time of the cells (Table 1). The cells were observed every day under an inverted phase microscope. Optimal confluence of the adherent cell monolayer for the performance of the experiment for evaluation of antiviral activity was achieved on the 24th hour after plating of 0.15 × 10^6^ cells/mL. Therefore, this initial cell number was used in all further experiments performed in 96-well plates. For other culture plates, the cell density was recalculated according to the culture surface and the volume of the sample in the well.

### 3.2. In Vitro Cytotoxicity of MB and TBO on MDBK Cells

**Figure 2:**
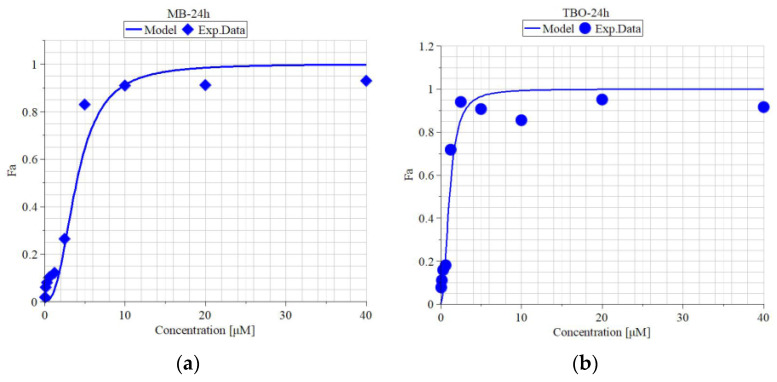

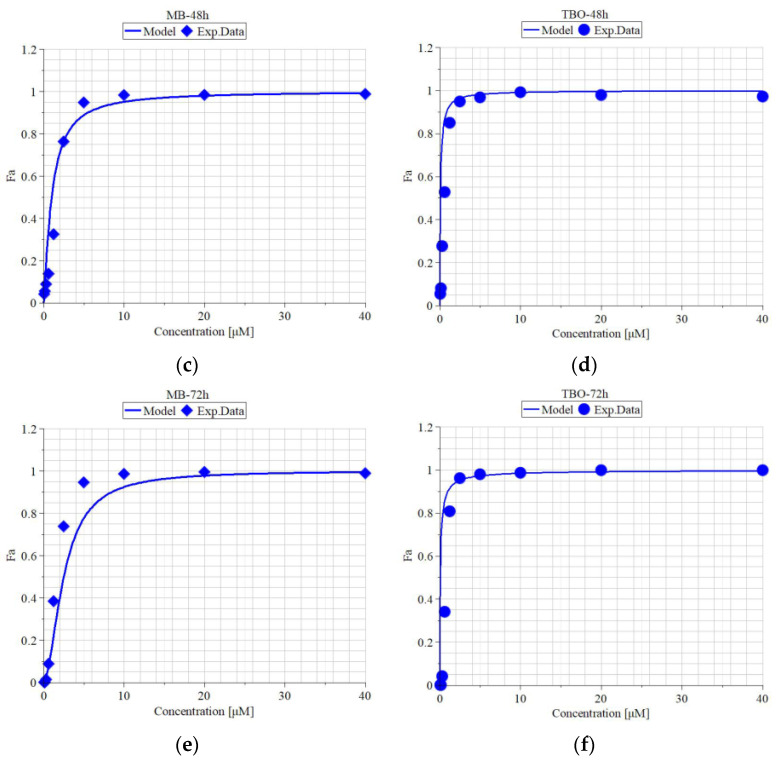
In vitro cytotoxicity of methylene blue and toluidine blue on MDBK cells after 24, 48 and 72 h exposure. Legend: Fa—antiviral activity (effect); MB—methylene blue; TBO—toluidine blue O; (**a**) Model and experimental data of Fa vs. concentration of MB after 24 h of exposure; (**b**) Model and experimental data of Fa vs. concentration of TBO after 24 h of exposure; (**c**) Model and experimental data of Fa vs. concentration of MB after 48 h of exposure; (**d**) Model and experimental data of Fa vs. concentration of TBO after 48 h of exposure; (**e**) Model and experimental data of Fa vs. concentration of MB after 72 h of exposure; (**f**) Model and experimental data of Fa vs. concentration of TBO after 72 h of exposure.

The in vitro cytotoxicity of MB and TBO on MDBK cells was evaluated based on the absorbance results from the MTT-dye reduction assay. Cells were treated for three incubation periods of time—24, 48, and 72 h—and the median inhibitory concentrations, as well as the maximal non-toxic concentrations were calculated in MAPLE 15^®^ software. The graphs with the “dose-effect” curves” are presented in Figure 2. As observed in the graphs, there is a concentration-dependent increase in the cytotoxic activity of the compounds.

The *IC*_50_ and MNC values, as well the hill slope “*m*” and the coefficient of determination “R^2^”, are given in Table 2. The *IC*_50_ of MB was between 1.28 and 2.54 µM, but the decrease was not time-dependent, as the *IC*_50_ after 48 h (1.28 µM) of incubation was lower than that after 72 h (1.8 µM) of incubation. The *IC*_50_ of TBO was lower than that of MB, ranging between 0.52 and 0.97 µM, wherein, again, no time-effect relationship was found—the *IC*_50_ after 48 h (0.52 µM) of incubation was lower than that after 72 h (0.85 µM) of incubation. The MNC for MB varied between 1.1 and 2.14 µM, whereas for TBO, it ranged between 0.35 and 0.66 µM.

### 3.3. Quantitative Evaluation of Virus Titer

The virus titer of the stock solution used in the presented experiments was determined via ddPCR, TCID_50_ assay, and plaque assay.

**Figure 3:**
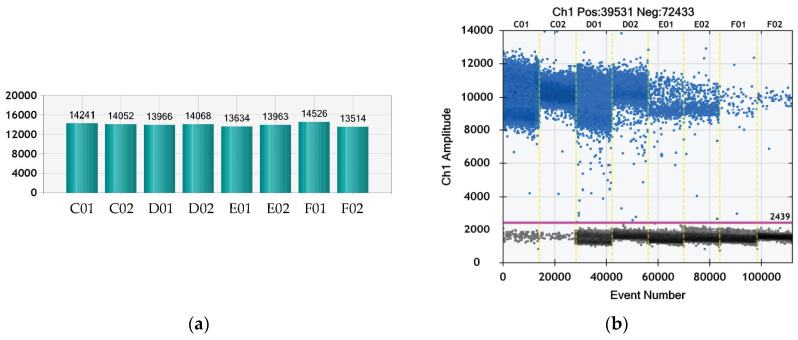
Histogram and total events from a ddPCR enumeration of the viral particles in the bovine coronavirus stock used for evaluation of PS antiviral activity. Legend: C01, C02 = dilution 10^−3^; D01, D02 = dilution 10^−4^; E01, E02 = dilution 10^−5^; F01, F02 = dilution 10^−6^.

In order to quantify the viral stock for the planned experiment, we performed, for the first time, ddPCR with the cited primer set using fluorophore (6-FAM) and quencher (BHQ1), which are suitable for the Bio-Rad ddPCR device. According to the ddPCR data presented in Figure 3 and in Table 3, the virus titer was 2.24 × 10^10^/mL. The number of generated droplets varied between 13,514 and 14,562 (Figure 3a), which is a marker for the reliability of the reaction, i.e., enough droplets were generated to ensure the statistical evaluation of the data and the calculation of the positive and negative events. The histogram in Figure 3b shows the positive fluorescent droplets and the negative no fluorescent droplets. The calculation of the cDNA concentration in each sample was performed with QuantaSoft^®^ software (Regulatory Edition #1864011, Laboratories Inc., Hercules, California, USA) supplied with the ddPCR device. The concentration of each dilution and repetition are given in Table 3. The median values were calculated based on the concentration of each repetition.

In parallel, a TCID_50_ assay was performed to determine the number of viable viral particles in the stock solution. According to calculations based on the methods of Reed-Muench and Kärber, the TCID_50_/mL was 3.48 × 10^8^. Based on this result, the PFU/mL was calculated to be 2.4 × 10^8^. The difference between the result from the TCID_50_ assay and that of the ddPCR could be due to the fact that ddPCR also detects viral RNA remnants from destroyed viral particles that do not cause infection of the cells [42].

**Figure 4:**
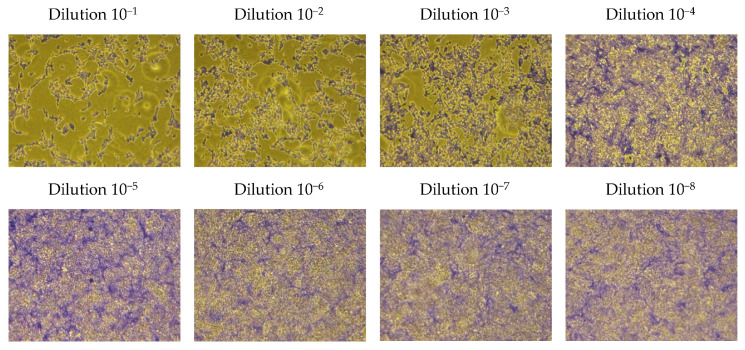
Micrographs from the plaque assay for determination of the virus titer, 200× magnification.

The microscopic pictures from the plaque assay applied for determination of the viral titer are presented in Figure 4. The plaques counted in dilution 10^−8^ were 2, pointing to a concentration of 2 PFU × 10^8^/mL. However, it was difficult to estimate the viral titer from the plaque assay, as no well-defined plaques were formed. In fact, upon increase of the dilution factor, the plaques became smaller in shape and more difficult to discern. Therefore, the result from the TCID_50_ assay was used in the subsequent experiments.

### 3.4. MB and TBO Inhibit the CPE of BCoV in MDBK Cells

**Figure 5:**
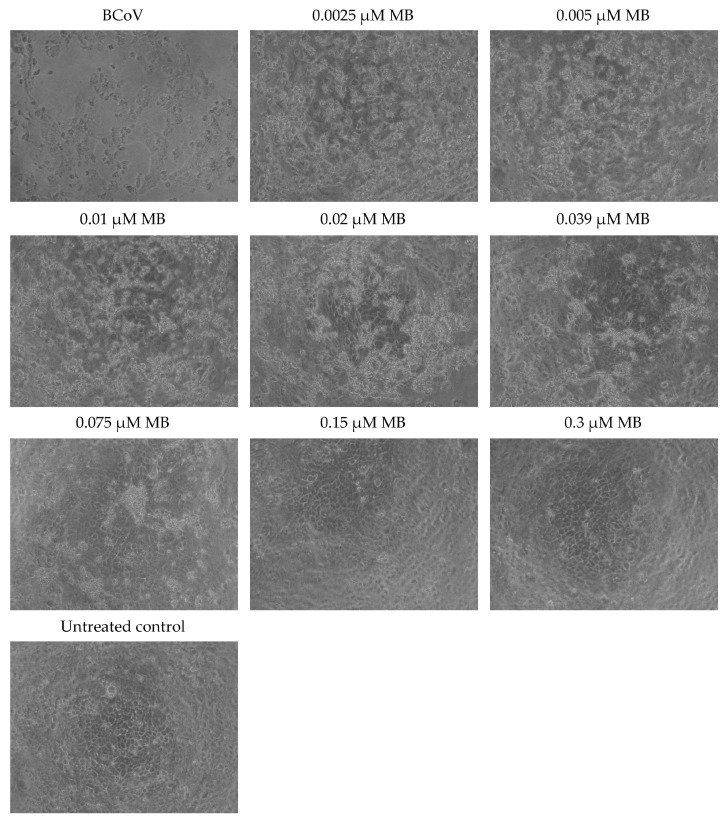
Microscopic observation of the cytopathic effect of BCoV on MDBK cells after direct inactivation with MB + irradiation—100× magnification.

**Figure 6:**
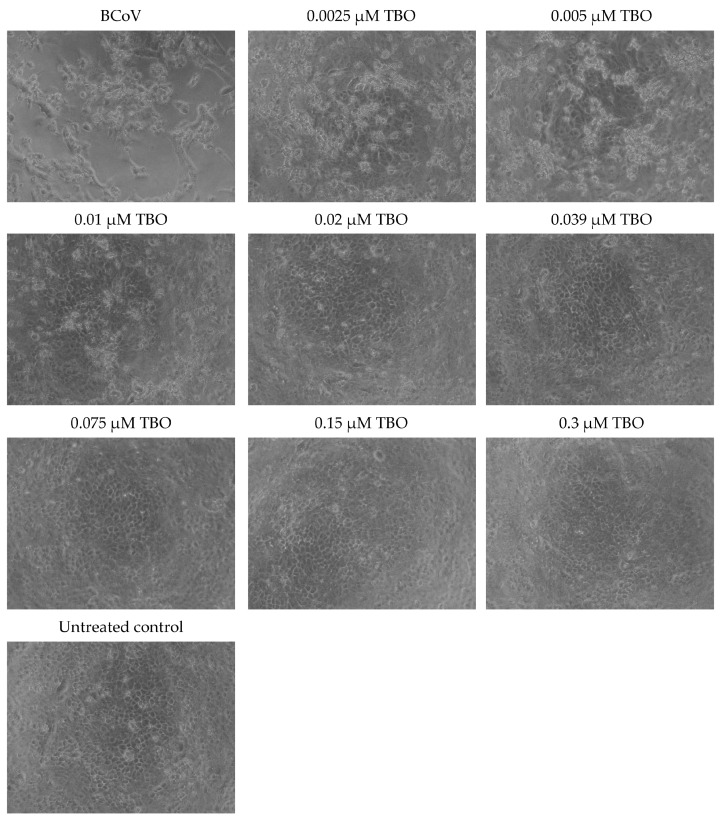
Microscopic observation of the cytopathic effect of BCoV on MDBK cells after direct inactivation with TBO + irradiation—100× magnification.

The CPE caused by the BCoV infection in MDBK cells, as well as its inhibition in samples treated with MB and TBO and irradiated with the respective light dose, is presented in Figure 5 and Figure 6. These figures demonstrate the changes in cellular morphology. Cell rounding, detachment, and clumping of adherent cells were observed in samples treated with low concentrations of MB (0.0025 µM, 0.005 µM, 0.01 µM, and 0.02 µM) and TBO (0.0025 µM and 0.005 µM). The cell density in these samples was uneven, with a small number of cells showing regular morphology and proportional cytoskeletal changes. In cultures treated with 0.039 µM and 0.075 µM of MB and 0.005 µM and 0.01 µM of TBO, the morphological signs corresponded to a picture of lower replication levels, whereby cells with morphology close to that of the untreated control began to proliferate. The cell density proportionally increases in a concentration-dependent manner and approaches the density of the untreated control. Manifestations of multipolarity were not observed and not counted. This sign is non-correlative because it may not be positive in cells with high proliferative potential. This leads to the preservation of their morphology for a long period of time, with an increase in their density or an increase in their concentration per unit area. The formation of plaque-forming units was greatly reduced in samples treated with MB and TBO, depending on the concentration used. Cells that were viable showed isolated rounding and, to a greater extent, clumping. These types of changes are indicative of cytoskeletal damage rather than the formation of new intercellular contacts and are presented in cultures treated with low concentrations of MB (0.002, 0.005, 0.01, and 0.02) and TBO (0.0025 and 0.005). In higher concentrations, stabilization of the culture, an increase in the intercellular contacts, and higher cell density in the monolayer are observed. Regarding the changes in external morphology, low concentrations of MB (0.0025, 0.005, 0.01, and 0.02 µM) and TBO (0.0025 and 0.005 µM) resulted in a small number of cells with preserved morphology but significant cell losses. These losses are demonstrated through destroyed cells and cell shadows (residual detritus). Regarding the signs for direct inactivation of the BCoV, in samples treated with 0.039 and 0.075 µM of MB and 0.005 and 0.01 µM of TBO, the cell concentration increases, and only individual plaque-forming units are present. Indirect markers of culture stabilization are the lack of cell migration and the increase in cell density, as observed in the samples treated with 0.039 and 0.075 µM for MB and 0.005 and 0.01 µM for TBO. The finding for permissive cells (cells with viral replication and cytopathic effects) was positive again in samples treated with a concentration of 0.039 and 0.075 µM for MB and 0.005 and 0.01 µM for TBO. The appearance of inadmissible cells (cells with no possibility of infection) is an effect that can be manifested in late stages and was not taken into account when observing the samples.

### 3.5. Metabolic Activity of MDBK Cells and Median Effective Concentrations of MB and TBO after Direct Inactivation of BCoV

**Figure 7:**
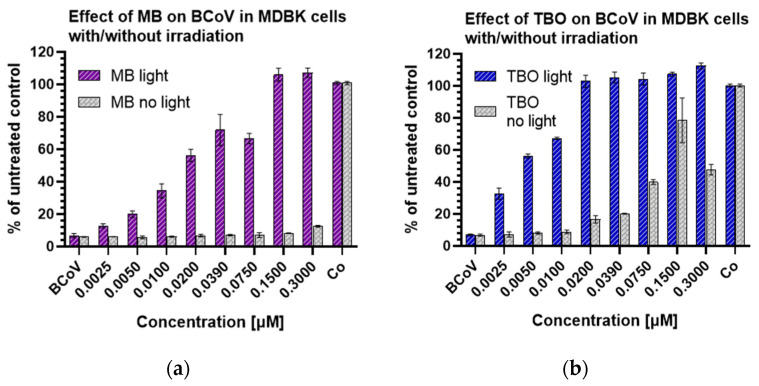
Evaluation of the viability of MDBK cells after exposure to MB or TBO in concentrations below the MNC: (**a**) comparison between the effects of MB with and without irradiation; (**b**) comparison between the effects of TBO with and without irradiation. Legend: MB—methylene blue; TBO—toluidine blue O; BCoV—bovine coronavirus; Co—untreated control.

The antiviral activity of MB and TBO was evaluated based on the data from the MTT assay after direct inactivation of the virus with the compounds and measurement of the metabolic activity of the cells in the tested samples. Figure 7 presents the cell viability of MDBK cells after direct inactivation of BCoV with MB or TBO, with or without light irradiation. Evidently, the irradiation-mediated antiviral activity of the photosensitizers is directly proportional to the applied concentration. The fraction of viable cells increases with the increase of the applied concentrations. When the treated samples were not irradiated, no antiviral activity was achieved. Approximately sevenfold lower concentration of TBO (0.02 µM) is enough to prevent the BCoV-induced metabolic inhibition of the cells, as compared to MB (0.15 µM).

**Figure 8:**
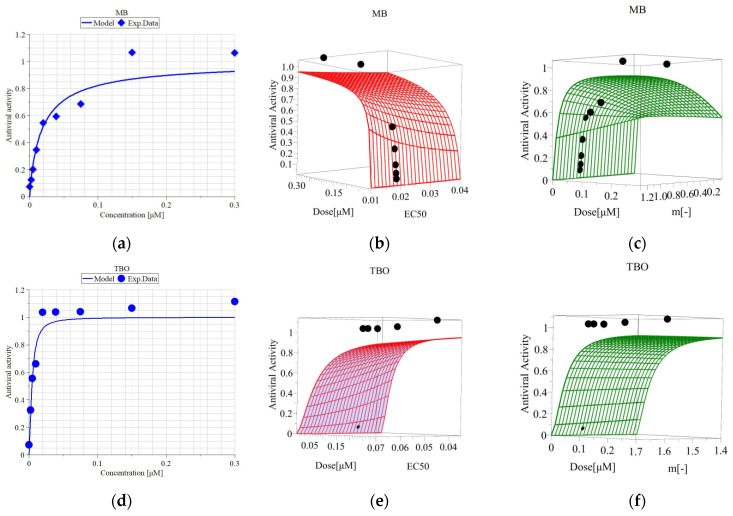
Antiviral activity of MB and TBO in MDBK cells after direct inactivation of BCoV with irradiation—non-linear curves for calculation of the median effective concentrations. Legend: MB—methylene blue; TBO—toluidine blue O; EC_50_—effective concentration 50%; *m*—hillslope; (**a**) Model and experimental data of antiviral activity (Fa) vs. applied concentration of MB; (**b**) Response surface analysis (RSA) of the antiviral activity of MB as a function of “Dose” (=concentration) and EC_50_, *m* = const.; (**c**) Response surface analysis (RSA) of the antiviral activity of MB as a function of “Dose” (=concentration) and *m*, EC_50_ = const.; (**d**) Model and experimental data of antiviral activity (Fa) vs. applied concentration of TBO; (**e**) Response surface analysis (RSA) of the antiviral activity of TBO as a function of “Dose” (=concentration) and EC_50_, *m* = const.; (**f**) Response surface analysis (RSA) of the antiviral activity of TBO as a function of “Dose” (=concentration) and *m*, EC_50_ = const.

The median effective concentrations (EC_50_) of both compounds, enough to inactivate the virus to a level resulting in 50% viable cells, were calculated from the curves presented in Figure 8. The RSA (Figure 8b,c,e,f) confirms the robustness and reliability of the model. The EC_50_ values and the parameters of the model are given in Table 4. The EC_50_ of TBO is 3.6-fold lower than that of MB, which is indicative for the significantly stronger activity of TBO.

The SI values for both compounds are given in Table 4. SI can be defined as the ratio of the toxic concentration of a compound against its effective bioactive concentration [41]. As calculated, the SI values for both compounds are higher than 10 (considered a “breakpoint” for suitability for further investigations [41]), with TBO having a higher SI than MB.

The statistical evaluation of the MTT data for the antiviral activity of MB and TBO are given in Table 5. The comparison of the treated samples reveals a significant difference between the effects of both compounds, indicating a significantly higher activity of TBO in the concentration range of 0.005–0.075 µM.

### 3.6. Quantitative Evaluation of the Virus Particles by ddPCR after Treatment with TBO

**Figure 9:**
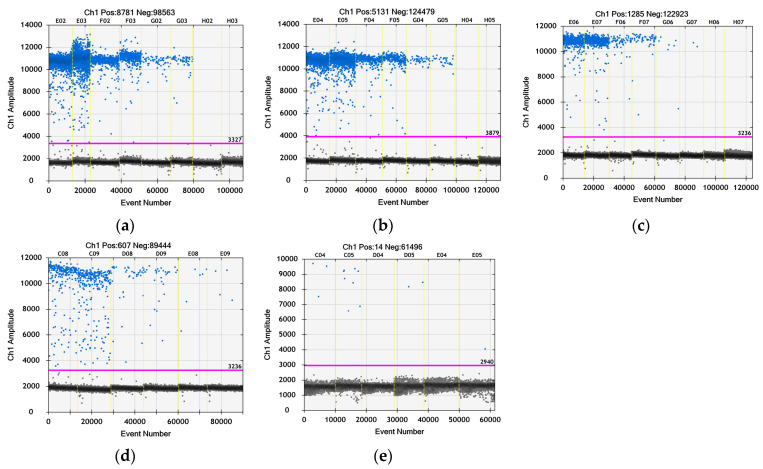
Histograms from a ddPCR assay for enumeration of the viral particles in three TBO-treated samples and the virus control—dilution of the cDNA. Legend: (**a**) BCoV (virus control)—E02, E03 = dilution 10^−4^; F02, F03 = dilution 10^−5^; G02, G03 = dilution 10^−6^; H02, H03 = non template control (PCR water); (**b**) TBO 0.005 µM—E04, E05 = dilution 10^−3^; F04, F05 = dilution 10^−4^; G04, G05 = dilution 10^−5^; H04, H05 = negative control from the RNA isolation; (**c**) TBO 0.02 µM –E06, E07 = dilution 10^−2^; F06, F07 = dilution 10^−3^; G06, G07 = dilution 10^−4^; H06, H07 = control with untreated cells without BCoV; (**d**) TBO 0.15 µM—C08, C09 = no dilution; D08, D09 = dilution 10^−1^; E08, E09 = dilution 10^−2^; (**e**) TBO 0.3 µM—C04, C05 no dilution; D04, D05 = dilution 10^−1^; E04, E05 = dilution 10^−2^.

The quantitative determination of the number of virus particles in the samples after exposure of BCoV to TBO was performed with ddPCR, and the results are presented in Figure 9 and in Table 6. The raw data are presented in Supplementary Figure S1 (total events in each sample prepared for ddPCR) and Supplementary Table S1 (concentration of BCoV cDNA in each repetition). A significant decrease in the number of virus particles, from 1.3 × 10^10^/mL in the virus control down to 2.2 × 10^3^/mL in the sample treated with 0.3 TBO, was found. The number of virus particles decreased inversely proportional to the applied concentration.

## 4. Discussion

In this study, we demonstrated, for the first time, the antiviral activity of the phenothiazine TBO on the replication of BCoV in vitro after direct inactivation approach. As a referent compound, we selected the well-studied MB dye and compared the potential of both for direct inactivation of BCoV. MB and TBO are both cationic photosensitizers. Recently, the existence of a negatively charged binding site for cationic PS at the connection of the S-protein stalk and the head adjacent to the HR2 domain was evidenced. This site is common for the S-proteins of SARS-CoV, SARS-CoV-2, and MERS-CoV [43], all of which belong to the same BCoV genus, Beta-coronavirus. This creates prospects for the wide use of this type of PS to combat the spread of coronaviruses.

It is reported in the literature that MB possesses so-called dual antiviral activity—with or without irradiation. The well-known MB light-induced activity finds its application for decontamination of plasma products in THERAFLEX MB-Plasma (Macopharma) [44]. MB has proven its potential to inhibit, independently of light, the replication of different viruses, e.g., SARS-CoV-2, the influenza virus H1N1 [45], Zika virus both in vitro and in vivo [46], and Dengue virus in plasma [47]. This activity also depends on the applied concentration; for example, Zhukhovitsky et al. observed this potential at a concentration ≥1 µM [48]. In our study, the highest tested concentration, 0.3 µM in the nontoxic concentration range for MDBK cells, was not enough to achieve “dark” antiviral activity towards BCoV (Figure 7a). The phenothiazine TBO investigated in our study was tested extensively for antibacterial activity [24,25,26] but not for antiviral activity against BCoV. It also possesses dual antiviral activity as MB but in much lower concentrations. The inhibition of the BCoV replication by TBO without irradiation in our experiment was well pronounced after applying 0.25 µM, which resulted in 80% viable cells (Figure 7b). Increasing the concentration applied without irradiation was already cytotoxic for the cell culture and diminished the fraction of viable cells by approximately 50% (Figure 7b). However, irradiation with the respective wavelength and J/cm^2^ (680 nm and 54 J/cm^2^ for MB; 635 nm and 99 J/cm^2^ for TBO) resulted in strong inactivation of BCoV in concentrations ≥ 0.5 µM for MB and 0.02 µM for TBO (Figure 7). Based on the values of the active concentrations, we determined that TBO is 25-fold more effective than MB. The EC_50_ values for both compounds—0.018 µM MB and 0.005 µM TBO—are also indicative of the stronger antiviral activity of TBO, namely, TBO is 3.6-fold more active than MB when it comes to 50% BCoV inactivation. Regarding the in vitro cytotoxicity on MDBK cells, we determined in our study that TBO possesses higher cytotoxicity than MB, expressed as two- to threefold lower *IC*_50_ for each incubation period of time. The *IC*_50_ value of TBO after 72 h of incubation was 2.12-fold lower than that of MB. However, the SI index (Table 4) of TBO (170) was 1.7-fold higher than that of MB (100). Our results for the antiviral activity of MB are in line with other studies, such as those by Zhukhovitsky et al. [48] and Arentz et al. [49]. The data on the antiviral activity of toluidine blue in the scientific literature are very scanty. The mode of action of this compound was discussed, together with that of MB and other cationic dyes, by Diederich et al. [50,51], who observed potentiation of the antiviral and IFN-inducing activities of dsRNA in L cells after exposure to such dyes. There is no detailed elucidation of this potentiated antiviral activity, but based on the fact that MB and TBO bind to RNA, an interaction of the dyes with the polyribonucleotide through intercalation was suggested [52]. A study by G.S. Thurner et al. [53] on the mode of action of the phenothiazine MB + PDI in vaccinia viruses demonstrated that 30 µM of MB leads to loss of virus infectivity, but not virus antigenicity, which makes PDT with MB a suitable approach for developing vaccines. Another mode of action of cationic photosensitizers discussed by Fedorov et al. is the ability of these dyes to bind to the S-protein stalk and the head adjacent to the HR2 domain of the SARS-CoV-2 virus, which represent areas of pronounced negative electrostatic potential [43]. It was suggested that this type of binding may interfere with the interaction of the S-protein with the ACE2 receptor of the host cell, thereby reducing virus infectivity. MB can directly damage the virus particles. The morphological evaluation of BCoV by Zhukhovitsky et al. under an electronic microscope after inactivation with 1 and 5 µM of MB revealed deterioration in the resolution of spikes, as well as a decrease in the size and disruption of the form of virus particles [48].

The CPE caused by the BCoV infection in MDBK cells in our study, as well as its inhibition in samples treated with MB and TBO and irradiated with the respective light dose, is indicative of the strong antiviral activity of MB and TBO on the bovine coronavirus. It is known from the scientific literature that virus-infected cells remain metabolically active for a certain period of time to support virus replication, and their pathomorphological changes are directly related to down-regulation of the expression of surface adhesion proteins as a result of the infection [54,55,56,57,58,59,60]. It was demonstrated in our experiments that such changes in cellular morphology, including cell rounding, detachment, and/or clumping of adherent cells, were observed after application of only lower concentrations of MB and TBO, in the range of 0.0025 µM to 0.02 µM for MB and 0.0025 µM to 0.005 µM for TBO. In cell samples treated with 0.039 µM and 0.075 µM of MB, or 0.005 µM and 0.01 µM of TBO, changes in cell morphology remained stable and clumps of adherent cells were limited in area. Higher concentrations of MB (0.15–0.3 µM) and TB (0.02–0.3 µM) were linked to cell morphology identical to untreated cells. Regarding the formation of plaque-forming units, the evaluation of morphological changes showed that it was greatly reduced in samples treated with MB and TBO, depending on the concentration used. For low concentrations of MB (0.002, 0.005, 0.01, and 0.02) and TBO (0.0025 and 0.005), the formation of intercellular contacts in the layer was still an isolated reaction. Intercellular contacts were preserved between and stabilized only within individual cells or areas of cells and were not repeatable in plaque-forming units. These changes seemed to be related to a higher level of cytopathicity and lower biological effectiveness of the photosensitizers applied. Application of a higher concentration of MB (>0.039 µM) and TBO (>0.01 µM) led to the stabilization of the culture and higher cell density in the monolayer, suggesting inhibition of the virus replication. It is well-known that changes in the external morphology of cells from the culture (cell curling) is a marker correlative to the loss of cells. Changes in the external morphology directly correlate with the manifested cytopathic effects and are proportional to cytoskeletal disorders. In this context, the change in the morphology of the nuclei is not an early sign of damage. This mark follows the general disturbances in the cytoskeleton and the integrity of the cell membranes. Such changes were observed in cell samples incubated with the lower range of concentrations of MB (0.0025, 0.005, 0.01, and 0.02 µM) and TBO (0.0025 and 0.005 µM). Increasing the concentrations up to 0.3 µM protected the cells from such changes in the external morphology, pointing to the assumption of virus particle inactivation after incubation with the photosensitizers in combination with the respective light irradiation. The signs of direct inactivation of the BCoV were the higher confluence of the culture monolayer, the preservation of intercellular contacts, and the decrease in cell losses and number of plaque-forming units in concentrations ≥0.039 µM of MB and ≥0.01 µM of TBO. This effect is demonstrated by a similar morphology with the untreated control. The presence of permissive and resolving cells also indicates stabilization of the culture and establishes a ratio between the cellular elements that become permissive cells (cells with viral replication and cytopathic effect) and resolving cells (cells with viral replication but no cytopathic effect). Permissive cells were observed again in samples treated with MB in concentrations up to 0.075 µM and with TBO in concentrations up to 0.01 µM. The cell morphology of samples incubated with MB concentrations higher than 0.075 µM of MB and 0.01 µM of TBO resembles that of the untreated control.

Finally, the ddPCR analysis of supernatants from TBO-treated samples (Figure 9, Table 6) confirmed the results obtained by the CPE estimation of MDBK cells (micrographs, Figure 5 and Figure 6) and the MTT assay (Figure 7). A clear and significant decrease in the number of viral cDNA (down to ~6 log/mL) was observed with an increase in the applied concentration of TBO.

All our findings confirm the antiviral activity of TBO against BCoV. TBO successfully inhibits the virus replication to a high extent and completely protects the cellular monolayer from the viral CPE. The high SI is indicative of the suitability of both dyes for use in veterinary and/or human medical practice. The recently published data from a clinical study with 1% MB for gargling or spraying in the nasal cavity demonstrated sufficient reduction of the SARS-CoV-2 virus load when applied in the early stages of the infection, resulting in significant decrease in morbidity and mortality. An advantage was the lack of adverse effects by local application even in patients with co-morbidities [61]. These results open new horizons for the future application of photosensitizers, i.e., TBO, for decontamination and prevention of coronavirus infections.

## 5. Conclusions

In conclusion, TBO directly inactivates, to a high extent, the bovine coronavirus in vitro after irradiation with 99 J/cm^2^ at 635 nm. Its antiviral activity is well pronounced at low concentrations, from 0.02 µM to 0.3 µM, which are not toxic to the MDBK cells, leading to complete protection of the cellular monolayer from the viral cytopathic effect. The selectivity index of TBO is very high (170), characterizing it as a selective bioactive compound which should be subjected to further pharmacological investigations aiming at its application in veterinary and/or human medical practice.

## Figures and Tables

**Table 1:** Absorbance of MDBK cells in culture used for calculation of the specific growth rate.

0 h			24 h		48 h		72 h		96 h		Doubling Time [h]
Cell Number	Abs	SD	Abs	SD	Abs	SD	Abs	SD	Abs	SD	
0.15 × 10^6^/mL	0.381	0.023	0.514	0.009	0.778	0.020	1.280	0.021	1.818	0.075	42.4
0.125 × 10^6^/mL	0.337	0.015	0.443	0.010	0.617	0.015	1.200	0.024	1.594	0.061	42.6
0.1 × 10^6^/mL	0.277	0.014	0.385	0.013	0.532	0.038	1.129	0.049	1.518	0.062	38.9
0.08 × 10^6^/mL	0.232	0.016	0.325	0.018	0.447	0.018	0.986	0.045	1.356	0.091	37.5
0.06 × 10^6^/mL	0.209	0.029	0.275	0.012	0.372	0.036	0.835	0.081	1.200	0.107	37.9
0.04 × 10^6^/mL	0.145	0.004	0.196	0.012	0.296	0.008	0.615	0.008	1.055	0.005	33.4

Legend: Abs—absorbance at 550 nm and ref. 690 nm (mean values from three measurements); SD—standard deviation.

**Table 2:** Median inhibitory and maximal nontoxic concentrations of MB and TBO in MDBK cells.

Parameters and Time of Incubation	Methylene Blue	Toluidine Blue O
24 h incubation:		
*IC*_50_ [µM]	2.54	0.97
Hill slope (m)	3.98	1.88
R^2^	0.99	0.95
MNC [µM]	2.14	0.66
48 h incubation:		
*IC*_50_	1.28	0.52
Hill slope (m)	1.00	0.098
R^2^	0.98	0.97
MNC [µM]	1.04	0.35
72 h incubation:		
*IC*_50_	1.80	0.85
Hill slope (m)	2.52	0.075
R^2^	0.98	0.96
MNC [µM]	1.1	0.59

Legend: *IC*_50_—median inhibitory concentration; R^2^—coefficient of determination; MNC—maximal nontoxic concentration.

**Table 3:** Concentration of the bovine coronavirus stock according to the ddPCR data analysis.

Sample	Concentration/20 µL *	Average Concentration/20 µL	Average Concentration of the Viral Stock/mL
C01 = dilution 10^−3^	1.07 × 10^5^	1.19 × 10^5^	1.06 × 10^8^/reaction (20 µL) ↓ 2.24 × 10^10^/mL **
C02 = dilution 10^−3^	1.31 × 10^5^		
D01 = dilution 10^−4^	1.1 × 10^4^	1.08 × 10^4^	
D02 = dilution 10^−4^	1.05 × 10^4^		
E01 = dilution 10^−5^	1 × 10^3^	0.94 × 10^3^	
E02 = dilution 10^−5^	0.88 × 10^3^		
F01 = dilution 10^−6^	1.2 × 10^2^	1.03 × 10^2^	
F02 = dilution 10^−6^	0.86 × 10^2^		

Legend: *—the volume of one ddPCR reaction is 20 µL; **—dilution factors and the method of calculation are given in section “Materials and methods”.

**Table 4:** Median and maximal effective concentrations of MB and TBO in MDBK cells after direct inactivation of BCoV with irradiation.

	EC_50_ [µM] (50% Viable Cells)	EC_100_ [µM] (100% Viable Cells)	Hill Slope	R^2^	SI (*IC*_50_/EC_50_)
**MB**	0.018	0.15	0.900	0.970	100
**TBO**	0.005	0.02	1.596	0.982	170

Legend: MB—methylene blue; TBO—toluidine blue O; EC_50_—effective antiviral concentration (50%) from the MTT based assay; R^2^—coefficient of determination; SI—selectivity index; *IC*_50_—inhibitory concentration (50%) from the in vitro cytotoxicity assay.

**Table 5:** Statistical evaluation and comparison of the antiviral activity of the tested concentrations of MB and TBO after direct inactivation of BCoV with irradiation.

Šídák’s Multiple Comparisons Test MB vs. TBO	Mean Difference	95.00% CI of Difference	Below Threshold?	Summary	“Adjusted *p* Value”
**BCoV**	0.01719	−5.908 to 5.942	No	ns	>0.9999
**0.0025**	−20.54	−26.47 to −14.62	Yes	****	<0.0001
**0.0050**	−35.60	−41.53 to −29.68	Yes	****	<0.0001
**0.0100**	−33.03	−38.96 to −27.11	Yes	****	<0.0001
**0.0200**	−48.96	−54.89 to −43.04	Yes	****	<0.0001
**0.0390**	−31.23	−37.15 to −25.30	Yes	****	<0.0001
**0.0750**	−36.42	−42.35 to −30.50	Yes	****	<0.0001
**0.1500**	−0.4070	−6.332 to 5.518	No	ns	>0.9999
**0.3000**	−6.087	−12.01 to −0.1619	Yes	*	0.0402
**Co**	0.01364	−5.912 to 5.939	No	ns	>0.9999

Legend: MB—methylene blue; TBO—toluidine blue O; BCoV—virus control with bovine coronavirus; vs.—versus; CI—confidence interval; ns—not significant; *—significant for *p* < 0.05; ****—significant for *p* < 0.0001; Co—untreated cell control (without BCoV and/or photosensitizers).

**Table 6:** Quantification of the bovine coronavirus particles in TBO-treated and untreated samples according to ddPCR data analysis.

Sample/Dilution of the cDNA	BCoV cDNA Concentration/Reaction *	BCoV cDNA Concentration/mL	Δlog cDNA/mL vs. Virus Control
Virus control:			
10^−4^	9.5 × 10^3^	1.3 × 10^10^	-
10^−5^	8.9 × 10^2^		
10^−6^	9.6 × 10^1^		
TBO 0.005 µM:			
10^−3^	3.5 × 10^3^	5.8 × 10^8^	2.24 × 10^1^
10^−4^	3.9 × 10^2^		
10^−5^	5.1 × 10^1^		
TBO 0.02 µM:			
10^−2^	9.2 × 10^2^	1.2 × 10^7^	1.08 × 10^3^
10^−3^	8.9 × 10^1^		
10^−4^	6.8 × 10^0^		
TBO 0.15 µM:			
-	4.3 × 10^2^	8.2 × 10^4^	1.59 × 10^5^
10^−1^	5.2 × 10^1^		
10^−2^	8.2 × 10^0^		
TBO 0.30 µM:			
-	1.4 × 10^1^	2.2 × 10^3^	5.91 × 10^6^
10^−1^	2.4 × 10^0^		
10^−2^	1.0 × 10^0^		

Legend: *—the volume of the ddPCR reaction is 20 µL; BCoV—bovine coronavirus; TBO—toluidine blue O; Δlog/mL—difference in the number of detected cDNA between the virus control and the respective sample treated with a certain concentration of TBO.

## Data Availability

All research data are available from the authors (see given e-mails).

## Supplementary Materials

The following supporting information can be downloaded at: https://www.mdpi.com/article/10.3390/v16010048/s1, Figure S1: Total events in the samples tested with ddPCR; Table S1: Concentrations of BCoV particles in the tested samples according to the ddPCR analysis—raw data of each repetition.
