# Supplementary material for: Photodynamic Inactivation of Bovine Coronavirus with the Photosensitizer Toluidine Blue O
Source: Viruses. 2023 Dec 27;16(1):48. doi: 10.3390/v16010048 (PMC10818719; doi:10.3390/v16010048)
Supplement: Supplementary file 1 [file viruses-16-00048-s001.zip › viruses-2770969-supplementary.pdf]

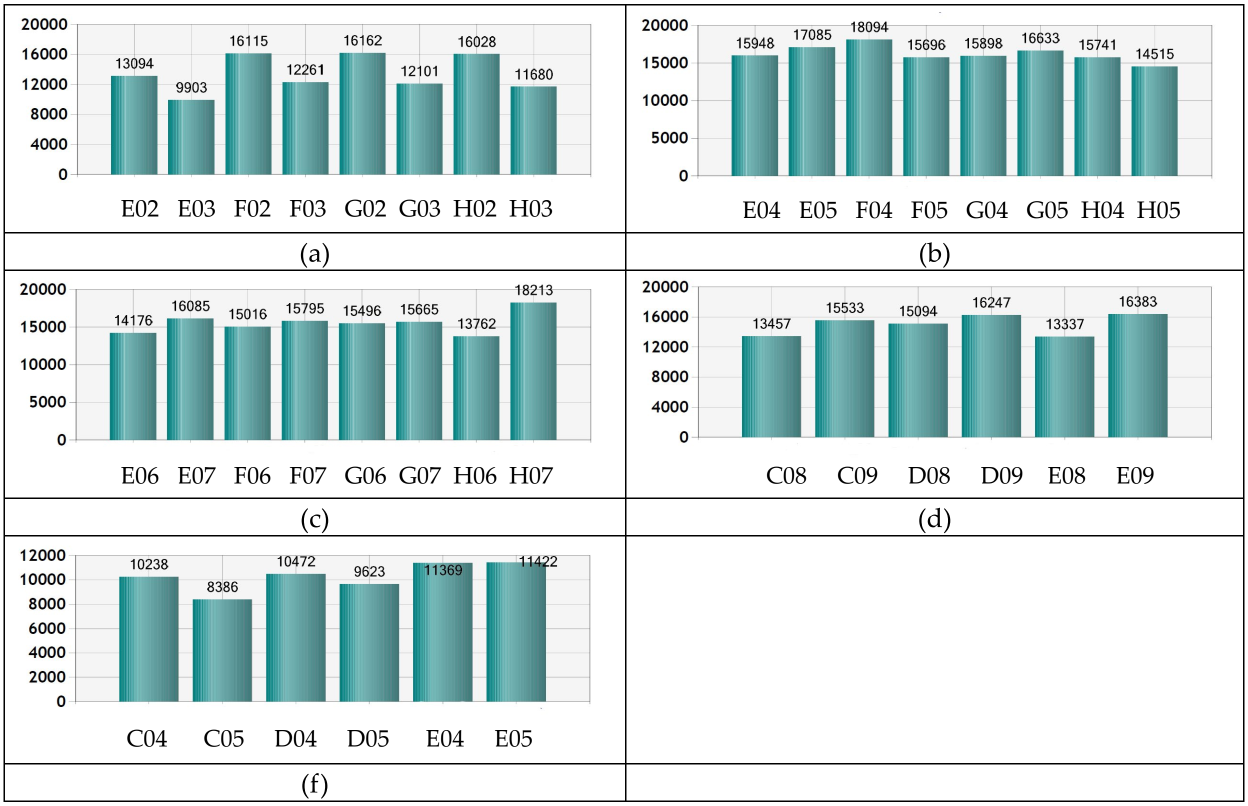

Supplementary Figure S1. Total events in the samples tested with ddPCR. Legend: (a) Cells with BCoV (virus control) – E02, E03 = dilution 10^{-4}; F02, F03 = dilution 10^{-5}; G02, G03 = dilution 10^{-6}; H02, H03 = non template control (PCR water); (b) TBO 0.005 μM – E04, E05 = dilution 10^{-3}; F04, F05 = dilution 10^{-4}; G04, G05 = dilution 10^{-5}; H04, H05 = negative control from the RNA isolation; (c) TBO 0.02 μM –E06, E07 = dilution 10^{-2}; F06, F07 = dilution 10^{-3}; G06, G07 = dilution 10^{-4}; H06, H07 = control with untreated cells without BCoV; (d) TBO 0.15 μM –C08, C09 = no dilution; D08, D09 = dilution 10^{-1}; E08, E09 = dilution 10^{-2}; (f) TBO 0.3 μM – C04, C05 no dilution; D04, D05 = dilution 10^{-1}; E04, E05 = dilution 10^{-2}.

Supplementary Table S1. Concentrations of BCoV particles in the tested samples according to the ddPCR analysis – raw data of each repetition.

**BCoV cDNA concentration/reaction* for each repetition**								
**Dilution**	**Virus control**	**Dilution**	**TBO 0.005 μM**	**Dilution**	**TBO 0.02 μM**	**Dilution**	**TBO 0.15 μM**	**TBO 0.3 μM**
10^{-4}	9.30×10^3	10^{-3}	3.54×10^3	10^{-2}	9.70×10^2	-	4.10×10^2	2.2×10^1
	9.64×10^3		3.36×10^3		8.74×10^2		4.52×10^2	6.8×10^0
10^{-5}	8.72×10^2	10^{-4}	8.72×10^2	10^{-3}	9.8×10^1	10^{-1}	6.0×10^1	4.8×10^0
	9.16×10^2		9.16×10^2		8.0×10^1		4.4×10^1	0.0×10^0
10^{-6}	8.6×10^1	10^{-5}	8.6×10^1	10^{-4}	1.06×10^1	10^{-2}	1.06×10^1	2.0×10^0
	1.06×10^2		1.06×10^2		3×10^0		5.8×10^0	0.0×10^0

Legend: * - the volume of the ddPCR reaction is 20 μL; TBO – toluidine blue O.
